# Uncharted

**DOI:** 10.1002/jhm.70212

**Published:** 2025-10-16

**Authors:** Julie Eckelbarger

**Affiliations:** ^1^ Wake Forest University School of Medicine, Department of Internal Medicine Winston‐Salem North Carolina USA

I am attending on the inpatient service. I round on a patient who I admitted 3 weeks ago, who has suffered complications while in the hospital, and is still on the service when I take back over after my week off. I know her husband, her daughter, and her two granddaughters by name. Her condition improves throughout the week. On Monday, I walk in and tell her today's the day she gets to go home. The atmosphere is joyous, near festive. She and her husband cry. The family asks for a picture with me, which I happily take, and it immediately gets sent to their family group chat. They thank me for taking such good care of the patient. Their praise is effusive. I am blushing. I am pleasantly overwhelmed.

No one will ever know of this happy moment. There is no record. There is no way to document the profound connection we made in this patient's difficult journey to discharge.

I go to the next patient, a man who is dying. I have spent most of the week discussing goals of care with him, his wife, and his brothers, who I know by name. We agree on discharge home with hospice to spend his remaining days surrounded by loved ones. Arrangements are made, and he also gets to be discharged home that day. Although the mood is different from the patient prior, the family and I share a sense of relief at accepting the next steps. Within hours of his discharge, I received a patient complaint to my email inbox. The niece of this patient, who was not at the bedside and not invited by the patient to the goals of care discussions, feels that the discharge to hospice was inappropriate, and improper medical care was provided. The only acceptable solution is a curative one.

I respond to emails and phone calls about this patient for the next 3 weeks. I recite the events and explain the decisions made again and again. The patient advocate and I are on a first‐name basis. The dissatisfaction with my actions is well‐documented and will be archived permanently.

I isolate these emails in a folder in my inbox called “patient care.”

I take sign‐out and prepare to go onto service again.

In medicine, moments that sustain us are often undocumented, while the moments that haunt us are preserved in perpetuity. We must carry the quiet victories in a ledger that no one sees while the loud misunderstandings are cc'd up the chain. There is no billing code for presence. No metric for trust. There is just the silent resolve to return to the bedside.

This emotional whiplash matters. Week after week, it compounds.

Apprehension grabbed hold of me in the lead‐up to my next week on service. I felt a looming sense of anticipatory dread that the next complaint I would spend weeks defending was lurking behind each door. When the shameful email chain lasts longer than the patient encounter, it is no surprise that the short‐lived positive interactions feel overshadowed. The sense that praise lives in fleeting memory while complaints live eternally in the chart is stifling.

I carry an ingrained concept from training around in my head: “if it's not documented, it didn't happen.” This dictum feels like a gross injustice as I grapple with these profound but uncharted interactions. While I can accept this phrase from a medico‐legal perspective, I find it dismissive of the humanistic side of medicine. Meaningful moments with patients do happen‐ moments that matter deeply to the patient and their care, even if they don't fit neatly into the ‘subjective, objective, assessment, plan’ “SOAP” note format.

Finding no outlet for this whiplash, I recorded my experience. I kept an unsent draft in my email, lodged in the “patient care” folder next to the frustrating record of the above complaint. Documenting the dueling good and bad allowed me to privately debrief in a system where I found no formal way to do so. Written months ago, this is a passage I find myself returning to, like a soothing balm that validates an invisible but common experience. So today, I share this story publicly with purpose. Intentional reflection is not the cure, nor is it preventative—but for me, it is a treatment. Good moments may be uncharted, but they are undeniably valuable. They justify being preserved, even if only for myself. Maybe even for the *Journal of Hospital Medicine*.

## CONFLICT OF INTEREST STATEMENT

The author declares no conflicts of interest.

